# Glucocorticoid Induces Incoordination between Glutamatergic and GABAergic Neurons in the Amygdala

**DOI:** 10.1371/journal.pone.0166535

**Published:** 2016-11-18

**Authors:** Guang-Yan Wang, Zhao-Ming Zhu, Shan Cui, Jin-Hui Wang

**Affiliations:** 1 Qingdao University, School of Pharmacy, 38 Dengzhou, Shandong, China; 2 State Key Lab of Brain and Cognitive Science, Institute of Biophysics, Chinese Academy of Sciences, Beijing, China; 3 University of Chinese Academy of Sciences, Beijing, China; Julius-Maximilians-Universitat Wurzburg, GERMANY

## Abstract

**Background:**

Stressful life leads to mood disorders. Chronic mild stress is presumably major etiology for depression, and acute severe stress leads to anxiety. These stressful situations may impair hypothalamus-pituitary-adrenal axis and in turn induce synapse dysfunction. However, it remains elusive how the stress hormones mess up subcellular compartments and interactions between excitatory and inhibitory neurons, which we have investigated in mouse amygdala, a structure related to emotional states.

**Methods and Results:**

Dexamethasone was chronically given by intraperitoneal injection once a day for one week or was acutely washed into the brain slices. The neuronal spikes and synaptic transmission were recorded by whole-cell patching in amygdala neurons of brain slices. The chronic or acute administration of dexamethasone downregulates glutamate release as well as upregulates GABA release and GABAergic neuron spiking. The chronic administration of dexamethasone also enhances the responsiveness of GABA receptors.

**Conclusion:**

The upregulation of GABAergic neurons and the downregulation of glutamatergic neurons by glucocorticoid impair their balance in the amygdala, which leads to emotional disorders during stress.

## Introduction

Major depression and anxiety are common psychiatric disorders, in which the dysfunctions of the neurons and synapses in the limbic system, such as amygdala, nucleus accumbens and prefrontal cortex, are presumably involved [1–14]. In terms of etiology for these emotional disorders, the physical and psychological stresses to the genetically susceptible individuals impair the functions of hypothalamus-pituitary-adrenal axis [15–19], and induce the neuron atrophy of the limbic system. To the influence of stress hormones on the functions of the synapses and neurons, previous studies indicate that glucocorticoid regulates the function of GABA_A_ receptors [20–22] and diminishes the density of GABAergic neurons in prenatal period [23]. Chronic stress deteriorates the reversal potentials and densities of GABA_A_ receptor-channels [24–26] and the strength of GABA synaptic transmission [27–30]. How stress hormones influence the subcellular compartments of the neurons as well as the interactions between excitatory and inhibitory neurons, i.e., cell-specific pathology, remains to be studied [31], since their coordination is critical for the neuronal encoding to manage well-organized cognitions [32, 33].

The amygdala, an important structure in the limbic system, is involved in the emotional processes including high and low mood states [34–39]. It plays a physiological role in fear memory [40–44]. Its dysfunction is associated to anxiety [5, 6, 45, 46] and major depressive disorder [47, 48]. Glucocorticoid receptors are localized in amygdala neurons [49]. As physiological interactions and balances between glutamatergic and GABAergic neurons are critical for maintaining normal brain functions [50], how glucocorticoids regulate these neurons and their interactions remains to be investigated in the amygdala [51].

In the present study, we aim to examine how glucocorticoids affect the subcellular compartments and interactions between glutamatergic and GABAergic neurons in the amygdala, which may be involved in the pathological processes of major depression and anxiety. Glucocorticoid influence was examined by acutely washing dexamethasone to the brain slices including amygdala or by chronically intraperitoneally injecting dexamethasone in the mice. GABAergic and glutamatergic neurons in these mice are genetically labeled by green and yellow fluorescent proteins, respectively, for cellular identity. Their excitability and synaptic transmission were measured by whole-cell recording at these neurons in the brain slices.

## Results

### Dexamethasone upregulates GABA release and GABA_A_ receptor responsiveness in the amygdala

**Fig 1:**
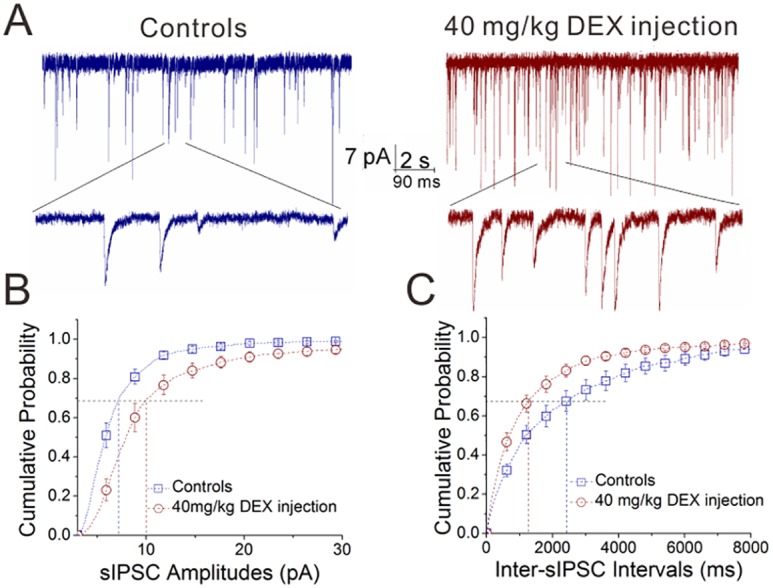
The chronic application of dexamethasone increases GABA release from inhibitory neurons and GABAergic receptor responses in excitatory neurons of the amygdala. DEX was used by intraperitoneal injections (40 mg/kg) per day for a week. sIPSCs were recorded in the glutamatergic neurons of the brain slices including the amygdala. **A**) shows sIPSCs in DEX injection (right panel) and saline injection (left). The calibration bars are 7 pA versus 2 seconds (top traces) and 90 ms (bottoms). **B**) illustrates cumulative probability versus sIPSC amplitudes in DEX injection (red symbols) and saline injection (blue). **C**) shows cumulative probability versus inter-sIPSC intervals in DEX injection (red symbols) and saline injection (blues).

The effect of dexamethasone, a synthetic glucocorticoid, on the action of the inhibitory neurons to the excitatory neurons was studied by recording sIPSCs on glutamatergic neurons in the mouse amygdala, in which the mice were treated by the intraperitoneal injections of dexamethasone (DEX) once a day (40 mg/kg) for a week. This chronic application of DEX appears to increase sIPSC frequency and amplitude (Fig 1A). Fig 1B shows cumulative probability versus sIPSC amplitude, in which sIPSC amplitudes at P_0.67_ are 10.21±1.09 pA in DEX injection (n = 11 cells from 8 mice) and 7.15±0.4 pA in saline injection (n = 13 cells from 6 mice, p<0.05). Fig 1C illustrates cumulative probability versus inter-sIPSC interval, in which inter-sIPSC intervals at P_0.67_ are 1337±216 ms in DEX injection (n = 11 cells from 8 mice) and 2741±511 ms in saline injection (n = 13 from 6 mice, p<0.05). The chronic application of dexamethasone increases GABA release from inhibitory neurons and GABA_A_ receptor responses on excitatory neurons in the amygdala.

**Fig 2:**
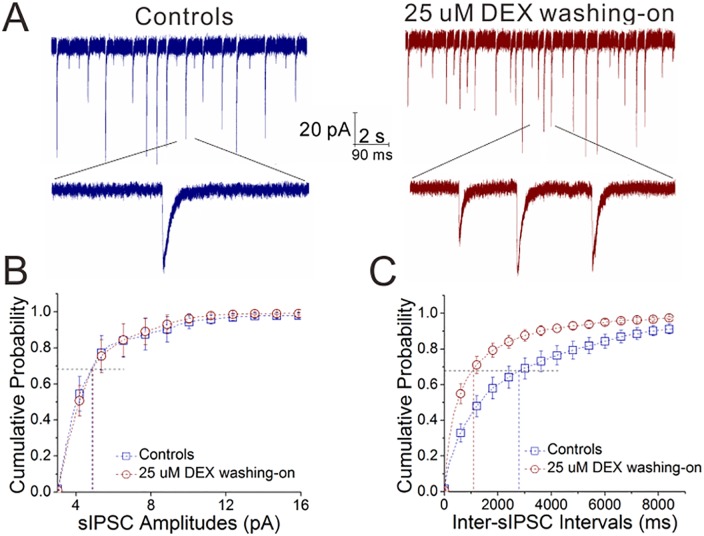
The acute application of dexamethasone increases GABA release from inhibitory neurons of the amygdala. DEX was administrated by washing into the brain slices (25 μM). sIPSCs were recorded in the glutamatergic neurons of brain slices including the amygdala. **A**) shows sIPSCs in the control (left panel) and DEX-washing (right). Calibration bars are 20 pA versus 2 seconds (top traces) and 90 ms (bottoms). **B**) shows cumulative probability versus sIPSC amplitude in the control (blue symbols) and DEX-washing (reds). **C**) illustrates cumulative probability versus inter-sIPSC intervals in the control (blue symbols) and DEX-washing (reds).

To examine the acute effect of dexamethasone, we washed DEX (25 μM) onto the brain slices in control mice. This acute application of DEX appears to increase sIPSC frequency (Fig 2A). Fig 2B shows cumulative probability versus sIPSC amplitudes, in which sIPSC amplitudes at P_0.67_ are 4.99±0.62 pA in the control and 5.0±0.56 pA after washing-on DEX (n = 10 cells from 8 mice). Fig 2C illustrates cumulative probability versus inter-sIPSC intervals, in which inter-sEPSC intervals at P_0.67_ are 2689±599 ms in the control and 1128±248 ms after washing-on DEX (n = 10 cells from 8 mice, p<0.05). The acute application of dexamethasone increases GABA release from inhibitory neurons in the amygdala.

### Dexamethasone upregulates the excitability of GABAergic neurons in the amygdala

**Fig 3:**
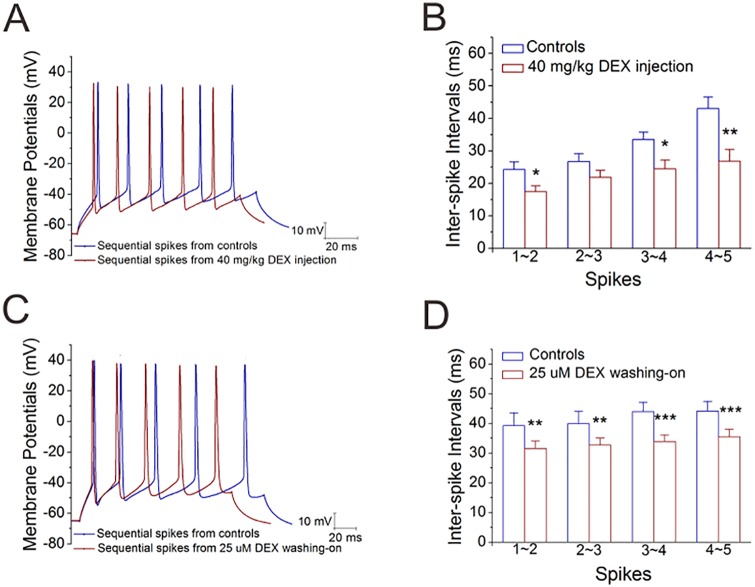
Dexamethasone increases the spike capability of inhibitory neurons in the amygdala. Sequential spikes were induced by depolarization pulses in the GABAergic neurons of the brain slices including the amygdala. **A~B)** DEX was used by intraperitoneal injections (40 mg/kg) per day for one week. **A**) shows sequential spikes in DEX injection (red trace) and saline injection (blue). Calibration bars are 10 mV versus 20 ms. **B**) illustrates inter-spike intervals versus number of spikes in DEX injection (red bars) and saline injection (blues). An asterisk is p<0.05 and two asterisks are p<0.01. **C~D)** DEX was administrated by washing into the brain slices (25 μM). **C**) shows sequential spikes in the control (blue trace) and DEX-washing (red). Calibration bars are 10 mV versus 20 ms. **D**) illustrates inter-spike intervals versus number of spikes in the control (blue bar) and DEX-washing (red). An asterisk is p<0.05, two asterisks are p<0.01 and three asterisks are p<0.001.

We studied whether DEX-induced increase in GABA release was due to an increased excitability in these inhibitory neurons, in which sequential spikes were induced by depolarization pulses (Methods). The chronic application of DEX by its intraperitoneal injection looks to enhance spike frequency (Fig 3A). Fig 3B illustrates that the intervals of spikes 1~2, 2~3, 3~4 and 4~5 are 17.48±1.73, 21.85±2.11, 24.5±2.63 and 26.76±3.68 ms in DEX injection (n = 11 cells from 6 mice) and 24.24±2.38, 26.71±2.43, 33.48±2.28 and 43.06±3.57 ms in saline injection (n = 11 cells from 6 mice, one asterisk, p<0.05 and two asterisks, p<0.01). Moreover, the acute application by washing DEX onto the brain slices appears to raise spike frequency (Fig 3C). Fig 3D illustrates that the intervals for spikes 1~2, 2~3, 3~4 and 4~5 are 39.17±4.39, 39.92±4.1, 43.92±3.2 and 44.1±3.37 ms in control and 31.52±2.5, 32.72±2.29, 33.85±2.3 and 35.53±2.45 ms after washing on DEX (n = 13 cells from 10 mice, two asterisks, p<0.01 and three asterisks, p<0.001). Thus, dexamethasone upregulates spike capability in the GABAergic neurons, which may lead to the increased GABA release in the amygdala.

### Dexamethasone downregulates glutamate release in the amygdala

**Fig 4:**
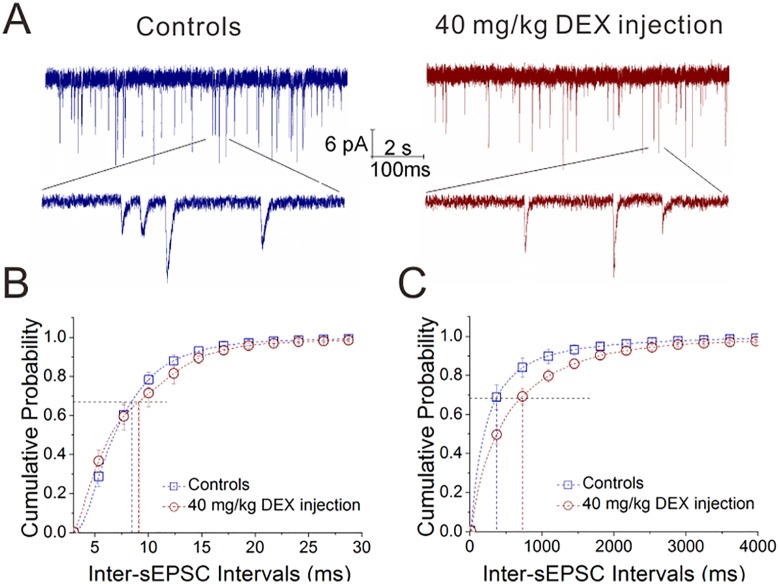
The chronic application of dexamethasone decreases glutamate release from excitatory neurons and increases glutamate receptor responses in the inhibitory neurons of the amygdala. DEX was used by intraperitoneal injections (40 mg/kg) per day for a week. sEPSCs were recorded in glutamatergic neurons of the brain slices including the amygdala. **A**) shows sEPSCs in DEX injection (right panel) and saline injection (left). The calibration bars are 6 pA versus 2 seconds (top traces) and 100 ms (bottom traces). **B**) shows cumulative probability vs. sEPSC amplitudes in DEX injection (red symbols) and saline injection (blue). **C**) shows cumulative probability versus inter-sEPSC intervals in DEX injection (red symbols) and saline injection (blue).

The effect of dexamethasone on the action of the excitatory neurons to the inhibitory neurons was studied by recording sEPSCs on the GABAergic neurons in the mouse amygdala, in which the mice were treated by intraperitoneal injections of DEX once a day (40 mg/kg) for one week. This chronic application of DEX appears to reduce sEPSC frequency (Fig 4A). Fig 4B demonstrates cumulative probability versus sEPSC amplitudes, in which sEPSC amplitudes at P_0.67_ are 8.53±0.79 pA in DEX injection (n = 19 cells from 6 mice) and 8.58±0.67 pA in saline injection (n = 12 cells from 6 mice, p = 0.9). Fig 4C illustrates cumulative probability versus inter-sEPSC intervals, in which inter-sEPSC intervals at P_0.67_ are 756±94 ms in DEX injection (n = 19 cells from 6 mice) and 428±101 ms in saline injection (n = 12 cells from 6 mice, p<0.05). Thus, the chronic application of dexamethasone downregulates glutamate release from excitatory neurons in the amygdala.

**Fig 5:**
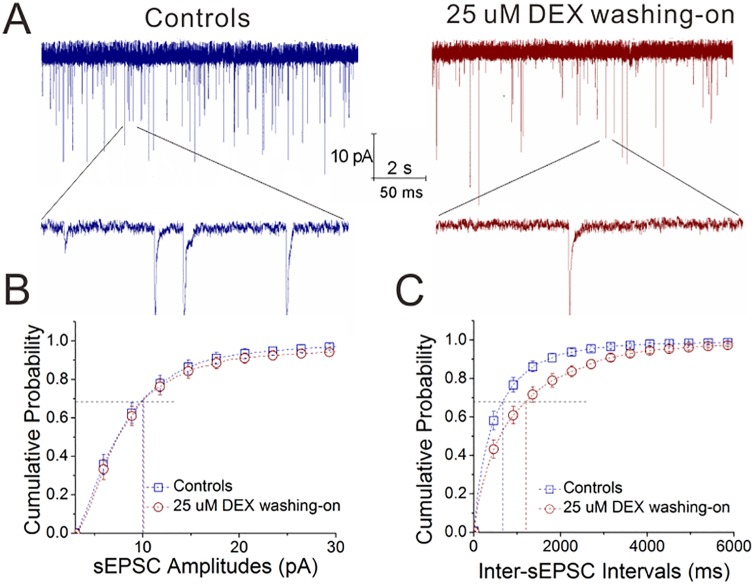
The acute application of dexamethasone decreases glutamate release from excitatory neurons of the amygdala. DEX was administrated by washing into the brain slices (25 μM). sEPSCs were recorded in the glutamatergic neurons of brain slices including the amygdala. **A**) shows sEPSCs in the control (left panel) and DEX-washing (right). The calibration bars are 10 pA versus 2 seconds (top traces) and 50 ms (bottoms). **B**) illustrates cumulative probability versus sEPSC amplitude in the control (blue symbols) and DEX-washing (red). **C**) illustrates cumulative probability versus inter-sEPSC intervals in the control (blue symbols) and DEX-washing (red).

To examine this result, we washed DEX (25 μM) onto the brain slices in control mice. This acute application of DEX appears to decrease sEPSC frequency (Fig 5A). Fig 5B illustrates cumulative probability versus sEPSC amplitudes, in which sEPSC amplitudes at P_0.67_ are 10.1±1.1 pA in the control and 10.42±0.93 pA after washing DEX (n = 14 cells from 10 mice, p = 0.8). Fig 5C shows cumulative probability versus inter-sEPSC interval, in which inter-sEPSC intervals at P_0.67_ are 697±105ms in control and 1242±173 ms after washing DEX (n = 14 cells from 10 mice, p<0.001). Thus, the acute application of dexamethasone decreases glutamate release from the excitatory neurons in the amygdala.

### Dexamethasone does not affect the excitability of glutamatergic neurons in the amygdala

**Fig 6:**
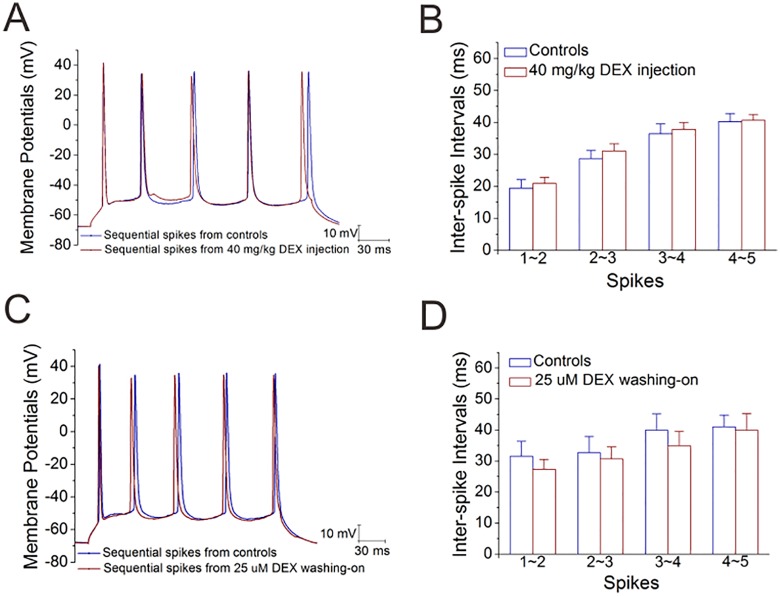
Dexamethasone does not affect spike abilities of excitatory neurons in the amygdala. Sequential spikes were induced by depolarization pulse in the glutamatergic neurons of the brain slices including the amygdala. **A~B)** DEX was used by intraperitoneal injections (40 mg/kg) per day for one week. **A**) shows sequential spikes in DEX injection (red trace) and saline injection (blue). Calibration bars are 10 mV vs. 30 ms. **B**) illustrates inter-spike intervals versus number of spikes in DEX injection (red bars) and saline injection (blues). An asterisk is p<0.05. **C~D)** DEX was administrated by washing into the brain slices (25 μM). **C**) shows sequential spikes in the control (blue trace) and DEX-washing (red). Calibration bars are 10 mV and 30 ms. **D**) illustrates inter-spike intervals versus number of spikes in the control (blue bar) and DEX-washing (red). An asterisk is p<0.05.

In the meantime, we examined whether DEX-induced downregulation in glutamate release is due to the decreased excitability in these glutamatergic neurons. As showed in Fig 6, inter-spike intervals are not changed by either chronic or acute application of DEX. Therefore, dexamethasone downregulates the subcellular compartment of glutamate release in the excitatory neurons from the amygdala.

## Discussion

**Fig 7:**
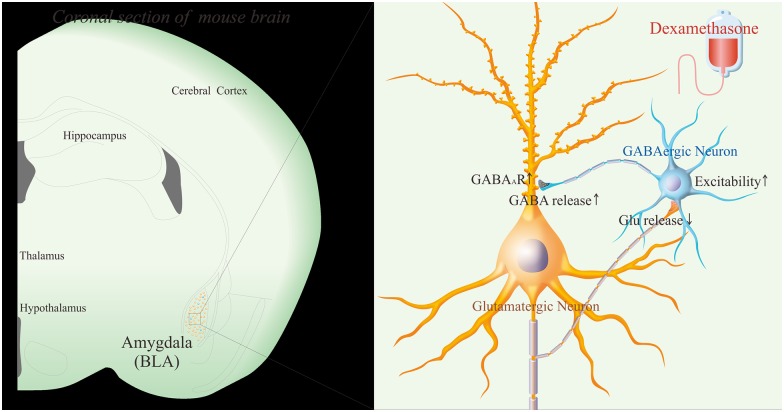
The differential effects of glucocorticoid on inhibitory and excitatory neurons in the basal lateral area (BLA) of the amygdala. Dexamethasone upregulates the action onto the glutamatergic neurons from GABAergic neurons by elevating presynaptic neuronal excitability, GABA release as well as postsynaptic GABA_A_ receptor responses. Dexamethasone downregulates the action onto the GABAergic neurons from glutamatergic neurons by lowering glutamate release. These alternations imbalance neural networks in the amygdala toward inhibitory state that may be involved in emotional disorders induced by stress hormones.

To the roles of glucocorticoid in regulating interactions between excitatory and inhibitory neurons, our studies demonstrate that dexamethasone upregulates GABA release from inhibitory neurons, GABA_A_ receptor responses on excitatory neurons and GABAergic neuron excitability (Figs 1–3). On the other hand, dexamethasone downregulates glutamate release from excitatory neurons (Figs 4 and 5). Taken our data together, we suggest that glucocorticoids lead to the upregulated action of the inhibitory neurons onto the excitatory neurons, as well as the downregulated action of the excitatory neurons onto the inhibitory neurons. The imbalanced interaction between the excitatory and inhibitory neurons toward inhibitory state in neural networks of the amygdala (Fig 7) may be involved in emotional disorders induced by stress hormones.

Previous studies demonstrate that the chronic application of corticosterone impairs hippocampal neurogenesis leading to mood disorders [52, 53] and the acute application of dexamethasone facilitates GABAergic synaptic transmission in the hippocampus [21]. These studies have not compared the acute and chronic effects of glucocorticoids on neuronal activities as well as not examined the influences of glucocorticoids on neuronal activities in the amygdala. In order to address these issues, we have investigated the acute and chronic roles of glucocorticoids in regulating the interactions between excitatory and inhibitory neurons in the amygdala. Our studies suggest that the acute activation of glucocorticoid receptors targets onto the mechanisms relevant to the releases of GABA and glutamate in the presynaptic compartments; however, in addition to the releases of GABA and glutamate, the chronic activation of glucocorticoid receptors acts onto postsynaptic GABA_A_ receptors (Figs 1 and 2, 4 and 5). The mechanisms underlying these differences are being examined. In terms of the pathological impacts about the differential roles of acute and chronic glucocorticoid receptor activations in the neuron coordination, our explanation is given below. Acute severe stress usually leads to anxiety-related emotion and behaviors, such as posttraumatic stress disorder, while chronic mild stress with lack of rewards cause major depressive disorder. Our studies about the differential effects of acute and chronic dexamethasone application on GABA and glutamate release and receptor responses may help to explain the consequences that chronic mild stress causes major depression and acute severe stress leads to anxiety-related disorders.

Our study first shows the different effects of glucocorticoid receptor activation on excitatory and inhibitory neurons. Dexamethasone upregulates spiking ability in GABAergic neurons (Fig 3), but not in glutamatergic neurons (Fig 6). Dexamethasone increases GABA release (Figs 1 and 2) and lowers glutamate release (Figs 4 and 5). Thus, the activation of glucocorticoid receptors may shift the balances between excitatory and inhibitory neurons in the amygdala, which is involved in stress-induced emotional disorders. In addition, our data imply that glucocorticoid receptors, their intracellular signaling pathways and their targeted receptor-channels in the excitatory versus inhibitory neurons may be different, which is worthy to be deeply studied in the field of neuroscience.

Our studies also indicate that the different subcellular compartments of excitatory and inhibitory neurons express differential sensitivity to glucocorticoid action. For instance, the presynaptic terminals of glutamatergic neuron are sensitive to dexamethasone (Figs 4 and 5) but not their cell body (Fig 6). The presynaptic terminals of excitatory and inhibitory neurons are sensitive to both acute and chronic actions of dexamethasone, but the postsynaptic GABA_A_ receptors in the neurons are only sensitive to the chronic action of dexamethasone (Figs 1, 2, 4 and 5). This incoordination among the subcellular compartments may lead to the complication of stress-induced emotional disorders [54].

By using mice with genetically YFP-labeled glutamatergic neurons and GFP-labeled GABAergic neurons in the amygdala, we are able to analyze cell-specific effect by glucocorticoids in their subcellular compartments and mutual interactions. The glucocorticoid-induced incompatibility among the subcellular compartments and the incoordination between GABAergic and glutamatergic neurons lead to imbalanced neural networks in the amygdala, which are the bases of stress-induced mood disorders.

## Methods and Materials

All experiments were done in accordance with the guideline and regulation by the Administration Office of Laboratory Animals at Beijing China. All experimental protocols were approved by Institutional Animal Care Unit Committee in Administration Office of Laboratory Animals at Beijing China (B10831).

### Acute and chronic applications of dexamethasone

In order to study the effects of dexamethasone (DEX), a potent agonist of glucocorticoid receptors, on the interactions between excitatory and inhibitory neurons, we applied C57 Thy1YFP/GAD1GFP mice whose GABAergic and glutamatergic neurons were genetically labeled by green fluorescent protein (GFP) and yellow fluorescent protein (YFP), respectively [55, 56]. In chronic application [52], DEX (40mg per kilogram of body weight) was intraperitoneally injected into these mice (postnatal day 20) once a day for one week. As body weights of these mice we used were about 20 grams on average, each mouse received 0.8 mg (i.e., 2 μM) DEX. This DEX concentration was close to its dosage that affected neuronal activities in a range of 0.5~5 uM [57]. In addition, we have measured the level of corticosterone in the mice that had received chronic unpredicted mild stress in day three about 300 ng/mL (i.e., 0.76 uM) [58]. Thus, DEX concentration used in our study was similar to that in chronic stress, and not over dosage. In acute application [21], DEX (25 μM) was washed onto the brain slices including the amygdala after the control data had been collected. This DEX concentration was based on a view that the concentration of corticosterone may be higher under acute stress than chronic stress [59]. In addition, we have found that 25 nM and 250 nM DEX are not effective on sEPSCs on GABAergic neurons in the brain slices. One could argue that that dexamethasone is much stronger than corticosterone. In terms of pharmacological effects of dexamethasone versus corticosterone on biological processes, it is true that synthetic dexamethasone is stronger than natural corticosterone to affect the cells in the peripherals, such as anti-inflammation. However, the effects of dexamethasone and corticosterone on neuronal activities are variable and comparable in terms of their efficacies and concentrations [57, 60, 61].

### Brain slices and neurons

To have more health brain cells for whole-cell recordings, we prepared cortical slices by the following procedures. The mice were anesthetized by isoflurane inhaling, and were infused by the artificial cerebrospinal fluid (ACSF) and oxygenated (95%O_2_ and 5%CO_2_) at 4°C into their left ventricles until the bodies became cold, in which the concentrations (mM) of the chemicals were 124 NaCl, 3 KCl, 1.2 NaH_2_PO_4_, 26 NaHCO_3_, 0.5 CaCl_2_, 4 MgSO_4_, 10 dextrose and 220 sucrose at pH 7.35. The mouse heads were immediately decapitated by guillotine and placed into this cold oxygenated ACSF for the brain isolation [31]. The cortical slices (300 μm) in coronal direction were cut by Vibratome in this cold oxygenated ACSF. They were held in another oxygenated ACSF (124 NaCl, 3 KCl, 1.2 NaH_2_PO_4_, 26 NaHCO_3_, 2 CaCl_2_, 2 MgSO_4_, 10 dextrose, and 5 HEPES, pH 7.35) at 25°C for 2 hours. Each slice was then placed into a submersion chamber (Warner RC-26G) that was perfused by the oxygenated ACSF at 31°C for the electrophysiological recordings [62–64]. The chemical reagents were from Sigma.

Whole-cell recording was done on GFP-labeled GABAergic and YFP-labeled glutamate neurons in the basal lateral area of the amygdala under DIC-fluorescent microscope (Nikon FN-E600, Japan). The wavelength at 488 nm excited the fluorescence of GFP-labeled neurons, and that at 575 nm excited the fluorescence of YFP-labeled neurons. The GABAergic neurons expressed fast spikes with less adaptation in their amplitudes and frequencies, the typical properties for the interneurons [65–69]. The glutamatergic neurons showed the pyramidal somata and spike adaptation [31].

### Whole-cell recording and neuronal functions

The neurons were recorded by a MultiClamp-700B amplifier under voltage-clamp for their synaptic activity and the current-clamp for their intrinsic property. Electrical signals were inputted to the pClamp-10 (Axon Instrument Inc.) for data acquisition and analysis. An output bandwidth of this amplifier was set at 3 kHz [63]. The pipette solution to record excitatory activities included (mM) 150 K-gluconate, 5 NaCl, 5 HEPES, 0.4 EGTA, 4 Mg-ATP, 0.5 Tris-GTP, and 5 phosphocreatine (pH 7.35; [70, 71]). The solution to study inhibitory synapses contained (mM) 130 K-gluconate, 20 KCl, 5 NaCl, 5 HEPES, 0.5 EGTA, 4 Mg-ATP, 0.5 Tris–GTP and 5 phosphocreatine (pH 7.35; [46]). The pipette solutions were made freshly and filtered (0.1 μm). The osmolarity was 295~305 mOsmol and pipette resistance was 5~6 MΩ.

The functions of GABAergic neurons were studied including their active intrinsic properties and inhibitory outputs [72]. Their inhibitory outputs were assessed by recording spontaneous inhibitory postsynaptic currents (sIPSC) on glutamatergic neurons in the presence of 10 μM 6-Cyano-7-nitroquinoxaline-2,3-(1H,4H)-dione (CNQX) and 40 μM D-amino-5-phosphonovanolenic acid (D-AP5) in the ACSF to block ionotropic glutamatergic receptors. 10 μM bicuculline was washed onto the slices at the end of experiments for blocking sIPSCs to test that synaptic responses were mediated by GABA_A_R. The pipette solution with the high concentration of chloride ions makes reversal potential to be -42 mV. sIPSCs are inward when the membrane potential is held at -65 mV [46, 58, 73].

The functions of glutamatergic neurons were studied including the active intrinsic properties and excitatory outputs [72]. Their excitatory outputs were assessed by recording spontaneous excitatory postsynaptic currents (sEPSC) on GABAergic neurons in the presence of 10 μM bicuculline in the ACSF to block GABA_A_R [69, 72]. 10 μM CNQX and 40 μM D-AP5 were added into the ACSF at the end of experiments to test whether synaptic responses were mediated by glutamate receptor, which blocked sEPSCs in our studies.

The recording of spontaneous synaptic currents, instead of the evoked synaptic currents, is based on the following reasons. sEPSC and sIPSC amplitudes represent the responsiveness and the densities of postsynaptic receptors. The frequencies imply the probability of transmitter release from an axon terminal and the number of presynaptic axons innervated on the recorded neuron [74, 75]. These parameters can be used to analyze presynaptic and postsynaptic mechanisms about the neuronal interaction. The evoked postsynaptic currents cannot separate these mechanisms. We did not add TTX in the ACSF to record miniature postsynaptic currents as we had to record neuronal excitability. As the frequency of synaptic activities was less than those of sequential spikes and the spontaneous spikes were never recorded on the neurons in our cortical slices, sIPSCs and sEPSCs were not generated from spontaneous action potential. The synaptic events in our recording are presumably miniature postsynaptic currents. This point is granted by a single peak of postsynaptic currents in our study [31].

Action potentials at the cortical neurons were induced by injecting the depolarization pulse. Their excitability was assessed by inter-spike intervals when the depolarization pulses were given [76]. We did not measure rheobase to show cellular excitability, as this strength-duration relationship was used to estimate the ability to fire single spike. We measured the capability of firing sequential spikes [62, 68].

Data were analyzed if the recorded neurons had the resting membrane potentials negatively more than -60 mV, and action potential amplitudes more than 90 mV. The criteria for the acceptance of each experiment also included less than 5% changes in resting membrane potential, spike magnitude, and input resistance throughout each recording. The series and input resistances in all neurons were monitored by injecting hyperpolarization pulses (5 mV/50 ms), and calculated by voltage pulses versus instantaneous and steady-state currents. The values in the amplitudes and inter-event intervals of sIPSCs and sEPSCs were read at 67% of cumulative probability (P_0.67_) for their statistical comparisons [77]. It is noteworthy that the frequencies of sEPSCs and sIPSCs were applied to merit presynaptic transmitter release and the amplitudes of sEPSCs and sIPSCs were used to merit postsynaptic receptor functions [31].

### Statistical analyses

The data of electrophysiological studies are presented as mean±SEM. Based on the principle of statistics, the paired-t-test is used for the comparisons before and after administering drugs, physical/chemical stimulations or molecular manipulations in a given group, such that paired t-test is used for the statistical comparison in neural activities before and after washing dexamethasone. One-way ANOVA is routinely used for the comparisons between groups, such as drugs versus controls, such that one-way ANOVA is used to make statistical comparison in neural activities between dexamethasone injection and control groups. The criterion for statistical significance is set at *p<0*.*05*.

## References

[1] Banasr M, Dwyer JM, Duman RS. Cell atrophy and loss in depression: reversal by antidepressant treatment. Curr Opin Cell Biol. 2011;23(6):730–7. 10.1016/j.ceb.2011.09.002 21996102 PMC3259683

[2] Bennett P, Wilkinson C, Turner J, Brain K, Edwards RT, Griffith G, et al Psychological factors associated with emotional responses to receiving genetic risk information. Journal of genetic counseling. 2008;17(3):234–41. 10.1007/s10897-007-9136-x . 18259848

[3] Bishop SJ. Neurocongnitive mechanisms of anxiety: an integrative account. Trends in Cognitive Sciences. 2007;11(7):307–16. 10.1016/j.tics.2007.05.008 17553730

[4] Craske MG, Rauch SL, Ursano R, Prenoveau JP, D. S., Zinbarg RE. What is an anxiety disorder? Depression and Anxiety. 2009;26(12):1066–85. 10.1002/da.20633 19957279

[5] Davidson RJ. Anxiety and affective style: one of prefrontal cortex and amygdala. Biological Psychiatry. 2002;51(1):68–80. 11801232 10.1016/s0006-3223(01)01328-2

[6] Davis M, Walker DL, Miles L, Grillon C. Phasic vs sustained fear in rats and humans: role of the extended amygdala in fear vs. anxiety. Neuropsychopharmacology. 2010;35(1):105–35. 10.1038/npp.2009.109 19693004 PMC2795099

[7] Duman CH. Models of depression. Vitam Horm. 2010;82:1–21. 10.1016/S0083-6729(10)82001-1 . 20472130

[8] Elizalde N, Gil-Bea FJ, Ramirez MJ, Aisa B, Lasheras B, Del Rio J, et al Long-lasting behavioral effects and recognition memory deficit induced by chronic mild stress in mice: effect of antidepressant treatment. Psychopharmacology (Berl). 2008;199(1):1–14. 10.1007/s00213-007-1035-1 . 18470507

[9] Lin LC, Sibille E. Reduced brain somatostatin in mood disorders: a common pathophysiological substrate and drug target? Frontiers in pharmacology. 2013;4:110 10.3389/fphar.2013.00110 24058344 PMC3766825

[10] Ma K, Guo L, Xu A, Cui S, Wang JH. Molecular Mechanism for Stress-Induced Depression Assessed by Sequencing miRNA and mRNA in Medial Prefrontal Cortex. PLoS One. 2016;11(7):e0159093 10.1371/journal.pone.0159093 27427907 PMC4948880

[11] Pittenger C, Duman RS. Stress, depression, and neuroplasticity: a convergence of mechanisms. Neuropsychopharmacology. 2008;33(1):88–109. 10.1038/sj.npp.1301574 . 17851537

[12] Rickeles K, Rynn M. Overview and clinical presentation of generalized anxiety disorder. Psychiatric Clinicals of North America. 2001;24(1):1–17. 10.1016/s0193-953x(05)70203-3 11225502

[13] Sandi C, Haller J. Stress and the social brain: behavioural effects and neurobiological mechanisms. Nat Rev Neurosci. 2015;16(5):290–304. 10.1038/nrn3918 . 25891510

[14] Stein MB, Stein DJ. Social anxiety disorders. Lancet. 2008;371(9618):1115–25. 10.1016/S0140-6736(08)60488-2 18374843

[15] Berton O, Hahn CG, Thase ME. Are we getting closer to valid translational models for major depression? Science. 2012;338(6103):75–9. 10.1126/science.1222940 . 23042886

[16] Brunoni AR, Lopes M, Fregni F. A systematic review and meta-analysis of clinical studies on major depression and BDNF levels: implications for the role of neuroplasticity in depression. Int J Neuropsychopharmacol. 2008;11(8):1169–80. 10.1017/S1461145708009309 . 18752720

[17] Elhwuegi AS. Central monoamines and their role in major depression. Prog Neuropsychopharmacol Biol Psychiatry. 2004;28(3):435–51. 10.1016/j.pnpbp.2003.11.018 . 15093950

[18] Rohleder N, Wolf JM, Wolf OT. Glucocorticoid sensitivity of cognitive and inflammatory processes in depression and posttraumatic stress disorder. Neurosci Biobehav Rev. 2010;35(1):104–14. 10.1016/j.neubiorev.2009.12.003 . 20005894

[19] Strekalova T, Couch Y, Kholod N, Boyks M, Malin D, Leprince P, et al Update in the methodology of the chronic stress paradigm: internal control matters. Behavioral and brain functions: BBF. 2011;7:9 10.1186/1744-9081-7-9 21524310 PMC3111355

[20] Gunn BG, Brown AR, Lambert JJ, Belelli D. Neurosteroids and GABA(A) Receptor Interactions: A Focus on Stress. Front Neurosci. 2011;5:131 10.3389/fnins.2011.00131 22164129 PMC3230140

[21] Hu W, Zhang M, Czeh B, Flugge G, Zhang W. Stress impairs GABAergic network function in the hippocampus by activating nongenomic glucocorticoid receptors and affecting the integrity of the parvalbumin-expressing neuronal network. Neuropsychopharmacology. 2010;35(8):1693–707. 10.1038/npp.2010.31 20357756 PMC3055473

[22] Skilbeck KJ, Johnston GA, Hinton T. Stress and GABA receptors. J Neurochem. 2010;112(5):1115–30. 10.1111/j.1471-4159.2009.06539.x . 20002524

[23] Uchida T, Furukawa T, Iwata S, Yanagawa Y, Fukuda A. Selective loss of parvalbumin-positive GABAergic interneurons in the cerebral cortex of maternally stressed Gad1-heterozygous mouse offspring. Transl Psychiatry. 2014;4:e371 10.1038/tp.2014.13 24618690 PMC3966041

[24] MacKenzie G, Maguire J. Chronic stress shifts the GABA reversal potential in the hippocampus and increases seizure susceptibility. Epilepsy Res. 2015;109:13–27. 10.1016/j.eplepsyres.2014.10.003 25524838 PMC4272449

[25] Quintero L, Cardenas R, Suarez-Roca H. Stress-induced hyperalgesia is associated with a reduced and delayed GABA inhibitory control that enhances post-synaptic NMDA receptor activation in the spinal cord. Pain. 2011;152(8):1909–22. 10.1016/j.pain.2011.04.017 . 21636214

[26] Wislowska-Stanek A, Lehner M, Skorzewska A, Krzascik P, Maciejak P, Szyndler J, et al Changes in the brain expression of alpha-2 subunits of the GABA-A receptor after chronic restraint stress in low- and high-anxiety rats. Behav Brain Res. 2013;253:337–45. 10.1016/j.bbr.2013.07.042 . 23916758

[27] Hasler G, van der Veen JW, Tumonis T, Meyers N, Shen J, Drevets WC. Reduced prefrontal glutamate/glutamine and gamma-aminobutyric acid levels in major depression determined using proton magnetic resonance spectroscopy. Arch Gen Psychiatry. 2007;64(2):193–200. Epub 2007/02/07. 64/2/193. 10.1001/archpsyc.64.2.193 17283286

[28] Plante DT, Jensen JE, Schoerning L, Winkelman JW. Reduced gamma-aminobutyric acid in occipital and anterior cingulate cortices in primary insomnia: a link to major depressive disorder? Neuropsychopharmacology. 2012;37(6):1548–57. 10.1038/npp.2012.4 22318195 PMC3327859

[29] Seney ML, Tripp A, McCune S, Lewis D, Sibille E. Laminar and cellular analyses of reduced somatostatin gene expression in the subgenual anterior cingulate cortex in major depression. Neurobiol Dis. 2014;73C:213–9. 10.1016/j.nbd.2014.10.005 25315685 PMC4394026

[30] Torrey EF, Barci BM, Webster MJ, Bartko JJ, Meador-Woodruff JH, Knable MB. Neurochemical markers for schizophrenia, bipolar disorder, and major depression in postmortem brains. Biol Psychiatry. 2005;57(3):252–60. 10.1016/j.biopsych.2004.10.019 . 15691526

[31] Xu A, Cui S, Wang J. Incoordination among subcellular compartments is associated to depression-like behavior induced by chronic mild stress. International Journal of Neuropsychopharmacology. 2015; 10.1093/ijnp/pyv122 26506857 PMC4886664

[32] Ascoli GA, Alonso-Nanclares L, Anderson SA, Barrionuevo G, Benavides-Piccione R, Burkhalter A, et al Petilla terminology: nomenclature of features of GABAergic interneurons of the cerebral cortex. Nat Rev Neurosci. 2008;9(7):557–68. Epub 2008/06/24. nrn2402. 10.1038/nrn2402 18568015 PMC2868386

[33] Buzsaki G, Geisler C, Henze DA, Wang XJ. Interneuron Diversity series: Circuit complexity and axon wiring economy of cortical interneurons. Trends Neurosci. 2004;27(4):186–93. Epub 2004/03/30. 10.1016/j.tins.2004.02.007 S0166223604000645 [pii]. . 15046877

[34] Dityatev AE, Bolshakov VY. Amygdala, long-term potentiation, and fear conditioning. Neuroscientist. 2005;11(1):75–88. Epub 2005/01/06. 11/1/75. 10.1177/1073858404270857 15632280

[35] Gallagher M, Chiba AA. The amygdala and emotion. Current Opinion in Neurobiology. 1996;6(2):221–7. 8725964 10.1016/s0959-4388(96)80076-6

[36] Grundemann J, Luthi A. Ensemble coding in amygdala circuits for associative learning. Curr Opin Neurobiol. 2015;35:200–6. . 26531780 10.1016/j.conb.2015.10.005

[37] LeDoux J. The emotional brain, fear and the amygdala. Cellular and Molecular Neuroscience. 2003;23(4–5):727–38. 10.1023/A:1025048802629 PMC11530156 14514027

[38] Neugebauer V, Li W, Bird GC, Han JS. The amygdala and persistent pain. Neuroscientist. 2004;10(3):221–34. 10.1177/1073858403261077 15155061

[39] Swanson LW. The amygdala and its place in the cerebral hemisphere. Ann N Y Acad Sci. 2003;985:174–84. . 12724158 10.1111/j.1749-6632.2003.tb07081.x

[40] Ehrlich I, Humeau Y, Grenier F, Ciocchi S, Herry C, Luthi A. Amygdala inhibitory circuits and the control of fear memory. Neuron. 2009;62:757–71. 10.1016/j.neuron.2009.05.026 19555645

[41] Li H, Penzo MA, Taniguchi H, Kopec CD, Huang ZJ, Li B. Experience-dependent modification of a central amygdala fear circuit. Nat Neurosci. 2013;16(3):332–9. Epub 2013/01/29. nn.3322. 10.1038/nn.3322 23354330 PMC3581751

[42] Roozendaal B, McEwen BS, Chattarji S. Stress, memory and amygdala. Nature Reviews of Neuroscience. 2009;10(6):423–33. 10.1038/nrn2651 19469026

[43] Shumyatsky GP, Tsvetkov E, Malleret G, Vronskaya S, Hatton M, Hampton L, et al Identification of a signaling network in lateral nucleus of amygdala important for inhibiting memory specifically related to learned fear. Cell. 2002;111(6):905–18. Epub 2003/01/16. S0092867402011169 [pii]. . 12526815 10.1016/s0092-8674(02)01116-9

[44] Wolff SB, Grundemann J, Tovote P, Krabbe S, Jacobson GA, Muller C, et al Amygdala interneuron subtypes control fear learning through disinhibition. Nature. 2014;509(7501):453–8. 10.1038/nature13258 . 24814341

[45] Wu LJ, Ko SW, Toyoda H, Zhao MG, Xu H, Vadakkan KI, et al Increased anxiety-like behavior and enhanced synaptic efficacy in the amygdala of GluR5 knockout mice. PLoS ONE. 2007;2(1):e167 Epub 2007/01/25. 10.1371/journal.pone.0000167 17245443 PMC1766473

[46] Zhang F, Liu B, Lei Z, Wang J. mGluR1,5 activation improves network asynchrony and GABAergic synapse attenuation in the amygdala: implication for anxiety-like behavior in DBA/2 mice. Mol Brain. 2012;5(1):20. Epub 2012/06/12. 1756-6606-5-20. 22681774 10.1186/1756-6606-5-20 PMC3475049

[47] Guilloux JP, Douillard-Guilloux G, Kota R, Wang X, Gardier AM, Martinowich K, et al Molecular evidence for BDNF- and GABA-related dysfunctions in the amygdala of female subjects with major depression. Mol Psychiatry. 2012;17(11):1130–42. 10.1038/mp.2011.113 21912391 PMC3237836

[48] Karolewicz B, Szebeni K, Gilmore T, Maciag D, Stockmeier CA, Ordway GA. Elevated levels of NR2A and PSD-95 in the lateral amygdala in depression. Int J Neuropsychopharmacol. 2009;12(2):143–53. 10.1017/S1461145708008985 18570704 PMC2645479

[49] Johnson LR, Farb C, Morrison JH, McEwen BS, LeDoux JE. Localization of glucocorticoid receptors at postsynaptic membranes in the lateral amygdala. Neuroscience. 2005;136(1):289–99. 10.1016/j.neuroscience.2005.06.050 . 16181741

[50] Klausberger T, Somogyi P. Neuronal diversity and temporal dynamics: the unity of hippocampal circuit operations. Science. 2008;321(5885):53–7. Epub 2008/07/05. 321/5885/53. 10.1126/science.1149381 18599766 PMC4487503

[51] Capogna M. GABAergic cell type diversity in the basolateral amygdala. Curr Opin Neurobiol. 2014;26:110–6. 10.1016/j.conb.2014.01.006 . 24486420

[52] Yau SY, Lau BW, Tong JB, Wong R, Ching YP, Qiu G, et al Hippocampal neurogenesis and dendritic plasticity support running-improved spatial learning and depression-like behaviour in stressed rats. PLoS One. 2011;6(9):e24263 10.1371/journal.pone.0024263 21935393 PMC3174166

[53] Yau SY, So KF. Adult neurogenesis and dendritic remodeling in hippocampal plasticity: which one is more important? Cell Transplant. 2014;23(4–5):471–9. 10.3727/096368914X678283 . 24636187

[54] Wang J-H, Cui S. Multi-target therapy for subcellular incompatibility in brain disorders. Brain Disorders & Therapy. 2015;4(5):1–5. 10.4172/2168-975X.1000200

[55] Wang D, Zhao J, Gao Z, Chen N, Wen B, Lu W, et al Neurons in the barrel cortex turn into processing whisker and odor signals: a cellular mechanism for the storage and retrieval of associative signals. Front Cell Neurosci. 2015;9:320 10.3389/fncel.2015.00320 26347609 PMC4543922

[56] Zhang G, Gao Z, Guan S, Zhu Y, Wang JH. Upregulation of excitatory neurons and downregulation of inhibitory neurons in barrel cortex are associated with loss of whisker inputs. Mol Brain. 2013;6(1):2. Epub 2013/01/05. 1756-6606-6-2. 23286328 10.1186/1756-6606-6-2 PMC3548736

[57] Zhu MY, Wang WP, Bissette G. Neuroprotective effects of agmatine against cell damage caused by glucocorticoids in cultured rat hippocampal neurons. Neuroscience. 2006;141(4):2019–27. 10.1016/j.neuroscience.2006.05.011 16777341 PMC2921983

[58] Ma K, Xu A, Cui S, Sun M, Xue Y, Wang J-H. Impaired GABA synthesis, uptake and release are associated with depression-like behaviors induced by chronic mild stress. Translational Psychiatry. 2016;6(e910):1–10. 10.1038/tp.2016.181 27701406 PMC5315548

[59] Gong S, Miao YL, Jiao GZ, Sun MJ, Li H, Lin J, et al Dynamics and correlation of serum cortisol and corticosterone under different physiological or stressful conditions in mice. PLoS One. 2015;10(2):e0117503 10.1371/journal.pone.0117503 25699675 PMC4336318

[60] Suwanjang W, Holmstrom KM, Chetsawang B, Abramov AY. Glucocorticoids reduce intracellular calcium concentration and protects neurons against glutamate toxicity. Cell Calcium. 2013;53(4):256–63. 10.1016/j.ceca.2012.12.006 23340218 PMC4208294

[61] Yu IT, Lee SH, Lee YS, Son H. Differential effects of corticosterone and dexamethasone on hippocampal neurogenesis in vitro. Biochem Biophys Res Commun. 2004;317(2):484–90. 10.1016/j.bbrc.2004.03.071 . 15063783

[62] Chen N, Chen X, Wang J-H. Homeostasis established by coordination of subcellular compartment plasticity improves spike encoding. Journal of Cell Science. 2008;121(17):2961–71. 18697837 10.1242/jcs.022368

[63] Ge R, Qian H, Chen N, Wang JH. Input-dependent subcellular localization of spike initiation between soma and axon at cortical pyramidal neurons. Mol Brain. 2014;7(1):26 10.1186/1756-6606-7-26 . 24708847 PMC4022375

[64] Wang J-H, Kelly PT. Ca2+/CaM signalling pathway up-regulates glutamatergic synaptic function in non-pyramidal fast-spiking neurons of hippocampal CA1. J Physiol (Lond). 2001;533(2):407–22. 11389201 10.1111/j.1469-7793.2001.0407a.x PMC2278630

[65] Freund TF, Buzsaki G. Interneurons of the hippocampus. Hippocampus. 1996;6:347–470. 8915675 10.1002/(SICI)1098-1063(1996)6:4<347::AID-HIPO1>3.0.CO;2-I

[66] Lu W, Wen B, Zhang F, Wang JH. Voltage-independent sodium channels emerge for an expression of activity-induced spontaneous spikes in GABAergic neurons. Mol Brain. 2014;7(1):38 10.1186/1756-6606-7-38 24886791 PMC4039334

[67] McKay BE, Turner RW. Physiological and morphological development of the rat cerebellar Purkinje cell. Journal of Physiology (London). 2005;567(Pt3):829–50. 16002452 10.1113/jphysiol.2005.089383 PMC1474219

[68] Wang JH, Wei J, Chen X, Yu J, Chen N, Shi J. The gain and fidelity of transmission patterns at cortical excitatory unitary synapses improve spike encoding. Journal of Cell Science. 2008;121(17):2951–60. 18697836 10.1242/jcs.025684

[69] Yu J, Qian H, Wang JH. Upregulation of transmitter release probability improves a conversion of synaptic analogue signals into neuronal digital spikes. Mol Brain. 2012;5(1):26. Epub 2012/08/03. 1756-6606-5-26. 22852823 10.1186/1756-6606-5-26 PMC3497613

[70] Ge R, Qian H, Wang JH. Physiological synaptic signals initiate sequential spikes at soma of cortical pyramidal neurons. Mol Brain. 2011;4(1):19. 1756-6606-4-19. 21549002 10.1186/1756-6606-4-19 PMC3113741

[71] Yang Z, Gu E, Lu X, Wang JH. Essential role of axonal VGSC inactivation in time-dependent deceleration and unreliability of spike propagation at cerebellar Purkinje cells. Mol Brain. 2014;7:1 10.1186/1756-6606-7-1 24382121 PMC3880351

[72] Wang J-H. Short-term cerebral ischemia causes the dysfunction of interneurons and more excitation of pyramidal neurons. Brain Research Bulletin. 2003;60(1–2):53–8. 12725892 10.1016/s0361-9230(03)00026-1

[73] Wei J, Zhang M, Zhu Y, Wang JH. Ca2+-calmodulin signalling pathway upregulates GABA synaptic transmission through cytoskeleton-mediated mechanisms. Neuroscience. 2004;127:637–47. 10.1016/j.neuroscience.2004.05.056 15283963

[74] Stevens CF. Presynaptic function. Current Opinion in Neurobiology. 2004;14(3):341–5. 10.1016/j.conb.2004.04.004 15194114

[75] Zucker RS, Regehr WG. Short-term synaptic plasticity. Ann Rev Physiol. 2002;25:355–405. 10.1146/annurev.physiol.64.092501.114547 11826273

[76] Chen N, Chen SL, Wu YL, Wang JH. The refractory periods and threshold potentials of sequential spikes measured by whole-cell recordings. Biochemical and Biophysical Research Communications. 2006;340:151–7. 10.1016/j.bbrc.2005.11.170 16343428

[77] Wen B, Qian H, Feng J, Ge RJ, Xu X, Cui ZQ, et al A Portion of Inhibitory Neurons in Human Temporal Lobe Epilepsy are Functionally Upregulated: An Endogenous Mechanism for Seizure Termination. CNS Neurosci Ther. 2015;21(2):204–14. 10.1111/cns.12336 . 25475128 PMC6495511

